# Temporal Bone Osteomyelitis in a Child Closely Resembles Lateral Sinus Thrombosis: A Case Report

**Published:** 2018-07

**Authors:** David-Victor-Kumar Irugu, Madan Gupta, Prateek Sharma, Prashant-Pratap-Singh Ramteke, Suresh-Chandra Sharma

**Affiliations:** 1 *Department of Otorhinolaryngology and Head & Neck Surgery, All India Institute of Medical Sciences, India.*; 2 *Department of Pathology, All India Institute of Medical Sciences, India.*

**Keywords:** Endocarditis, Mucormycosis, Osteomyelitis, Osteoporosis, Osteopetrosis, Rhino-orbito-cerebral

## Abstract

**Introduction:**

Temporal bone osteomyelitis is more commonly seen in immunocompromised patients and is very rare in non-immunocompromised individuals. Mucormycosis is a fulminating fungal infection caused by Mucor which is a saprophytic fungus commonly seen in diabetic patients. Here we report a case of temporal bone osteomyelitis in a child with a traumatic history which was causing clinical features of lateral sinus thrombosis. The patient was successfully treated and doing well post-operatively.

**Case Report:**

An 11-year-old girl was reported to the emergency dept with fever and headache for 2 weeks. She had a fever of 100–102 °F without chills and rigors which was associated with severe headache on the right side and not associated with any vomiting, nausea, or aura. The patient did not have any other significant complaints except a history of falling 2 years previously when she was 9 years of age. The patient was admitted and a complete evaluation was performed clinically and radiologically. High-resolution computed tomography (HRCT) of the temporal bone was suggestive of soft tissue density at the sigmoid sinus of the right mastoid. The patient underwent surgery for debridement, and the tissue was sent for diagnosis. This revealed mucormycosis of the temporal bone and the patient started medical management. At the present date, the patient remains under follow up.

**Conclusion:**

Fungal chronic osteomyelitis is a disease among immune-compromised patients involving the temporal bone, and is very rare. In particular mucormycosis is very rare in the temporal bone but is not expected in normal individuals. HRCT of the temporal bone is the gold standard investigation, and tissue biopsy is diagnostic. Tissue debridement and long-time medical management with anti-fungal medication is mandatory to achieve good results.

## Introduction

Mucormycosis is an aggressive, opportunistic fungal infection (1). Mucor is a saprophytic fungus and causes aggressive angioinvasive disease in immunocompromised and diabetic patients (2,3). 

Mucormycosis presents in five principal forms: rhino-orbito-cerebral, pulmonary, disseminated, cutaneous, and gastrointestinal. In addition to these, mucormycosis can also cause endocarditis, osteomyelitis, peritonitis, and pyelonephritis which are less common. It rarely involves the ear or temporal bone, and there are few reports of invasive mucormycosis of the temporal bone in the literature (2,3).

Here we report a non-diabetic 11-year-old female patient who presented with fever and headache and was diagnosed with osteomyelitis (OM) of the temporal bone compressing the sigmoid sinus mimicking a lateral sinus thrombosis.

## Case Report

An 11-year-old girl was reported to ambulatory care with fever and headache for 2 weeks. Prior to these symptoms, the patient was in normal health. She had febrile episodes (100–102 °F) without chills or rigors. The fever was associated with severe headache on the right side and was not associated with any vomiting, nausea or aura. The patient did not have any other significant complaints, clinical signs or symptoms pertaining to the ear, nose, throat or chest, or bleeding diathesis except for a history of falling 2 years previously when she was 9 years of age.

We admitted the patient for further evaluation. On clinical examination, the patient had tenderness over the right mastoid bone with an intact tympanic membrane. With the above history, a provisional diagnosis of acute mastoiditis of the right side was made. Pediatric neurology, endocrinology, and ophthalmology consultations were obtained. On fundoscopy, the patient had bilateral papilledema with normal vision on both sides and was referred for further imaging with magnetic resonance imaging (MRI) of the brain which revealed no space-occupying lesions in the brain.

Routine investigations were all within normal limits except for erythrocyte sedimentation rate which showed a three-fold increase in the first hour. Subsequently the Mantoux test was performed which was positive (22-mm induration) and there was increased serum homocysteine.

Subsequently the patient was worked up for tuberculous OM, and a bone scan was performed for OM of the temporal bone. The patient had a positive Mantoux test (22-mm) and 99Tc- MDP triple phase bone scan with single-photon emission computed tomography (SPECT-CT) of the temporal bone which suggested asymmetric uptake with increased radiotracer accumulation in the right temporal bone posteriorly. The patient was kept on AKT but did not respond.

**Fig1:**
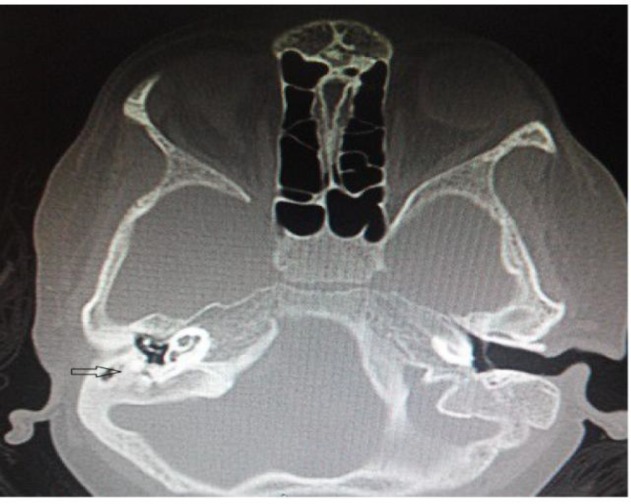
High-resolution computer tomography of temporal bone. Axial cuts showing cortical destruction with soft tissue density right side (Arrow

High-resolution computed tomography (HRCT) scan of the temporal bone showed mild sclerosis and cortical destruction involving the right temporal bone (Fig.1). With these findings, the patient was taken up for debridement to obtain tissue for histopathological diagnosis.

**Fig 2 (A):**
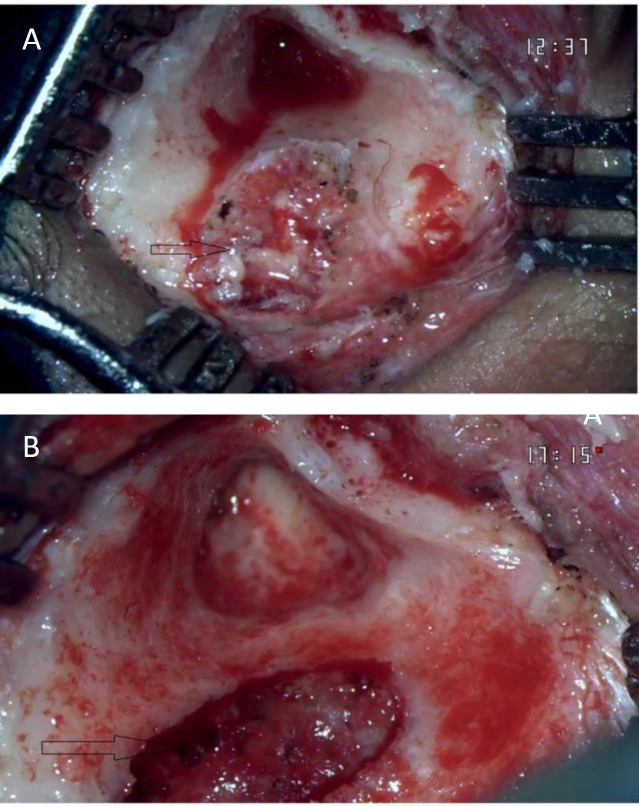
Intra-operative cheese-like material on sigmoid sinus with erosion of the lateral bony plate sigmoid sinus (Arrow).**(B): **Intra-operative picture after disease clearance with good flow in the sigmoid sinus (Arrow

The patient underwent right cortical mastoidectomy under general anesthesia. There was a mass with soft cheese-like material on the sigmoid sinus bony plate. The sigmoid sinus plate was eroded, and the mass was tracked medially and found to be compressing the lumen of the sigmoid sinus. The mass occupied the space between the sigmoid sinus bony plate and the membranous lumen, misleading the diagnosis as lateral sinus thrombosis (Fig.2 A,B). The disease was cleared overall and the sigmoid sinus was found to be normal with good flow as confirmed by needle aspiration.

**Fig 3 (A):**
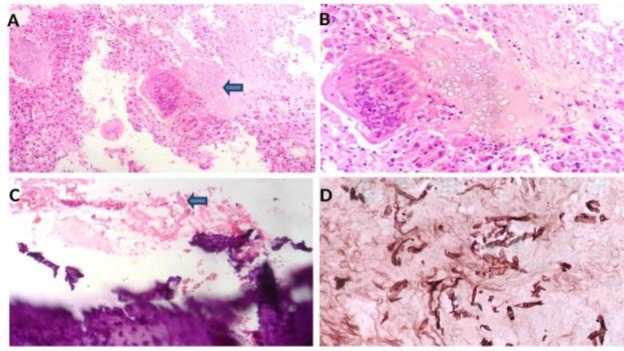
Hematoxylin and eosin, 200×, biopsy shows giant cell reaction, area of necrosis and giant cell containing fungal element. **(B):** Hematoxylin and eosin, 400×, giant cell containing transverse section of fungal elements. **(C):** Hematoxylin and eosin, 200×, bony tissue infiltrated by fungal element. **(D):** Silver methenamine stain, 400×, fibrous tissue infiltrated by wide, aseptate hyphae of a wide angle branched fungi morphologically compatible with Mucor species

Tissue was sent for histopathological examination which revealed Mucor mycosis-induced OM of the right temporal bone (Fig.3 A,B,C,D). Postoperatively, the patient received 2g cumulative dose of Amphotericin-B (liposomal) along with voriconazole 50mg per day for 3 weeks. 

**Fig 4:**
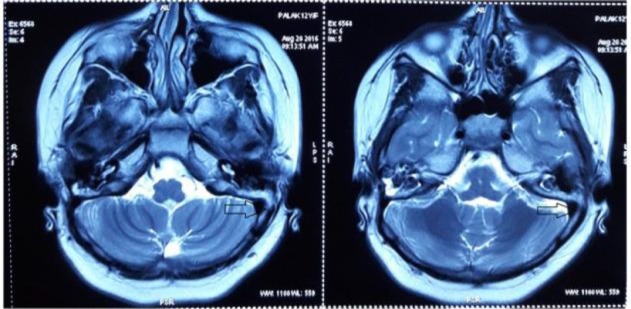
Post-operative MRI brain reveals mild narrowing seen right sigmoid sinus, transverse sinus with good contour (Arrow

**Fig 5:**
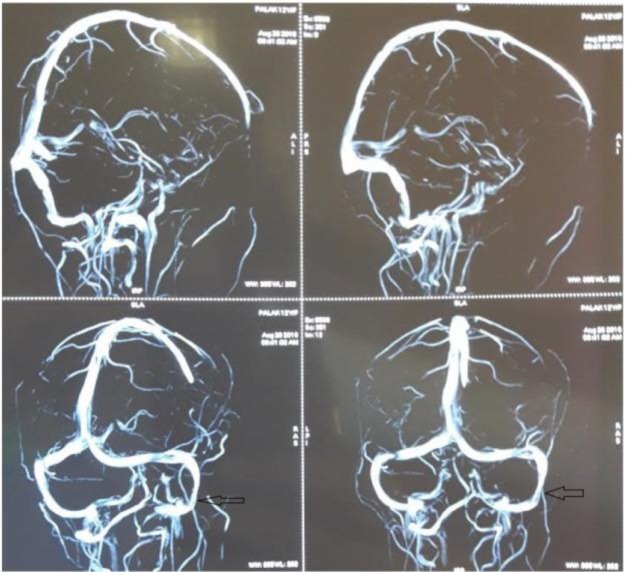
MRV reveals normal right sigmoid sinus with no evidence of thrombus or occlusion (Arrow)

The patient was strictly monitored for liver and renal function tests. The patient was free from symptoms after 3 weeks, with regular follow-ups to date. Post-operative magnetic resonance imaging (MRI) of the brain (Fig.4) and magnetic resonance venography (MRV) (Fig.5) after 6 months revealed post-canalised sigmoid sinus of the right side with good flow contour.

## Discussion

OM is a bone infection of the medullary cavity, rapidly involving the Haversian system and extends to involve the periosteum of the affected area (4). The term OM was coined by Nelaton in 1844, and most commonly occurs in long bones and vertebrae (4). The infection occurs because of bacteremia, inoculation of infective foci during surgery or trauma (4). In our case, trauma to the temporal bone may have introduced fungal foci which settled at the sigmoid plate in the right mastoid bone.

Predisposing factors like immunocompro- mised status, diabetes mellitus, increased age, anemia, malnutrition, altered blood supply to the bone, malignancy, osteoporosis, osteopetrosis and Paget’s disease increase the risk of OM (4,5). The most common etiological factors causing OM are Pseudomonas aeruginosa, Staphylococcus aureus, Proteus mirabilis, Klebsiella oxytocyta and fungi (6). The bones most commonly involved in the head and neck are the mandible, frontal bone, cervical spine, maxilla, nasal bones, temporal bone and skull base (4). In our case, it may be due to trauma to the temporal bone.

Mucormycosis is an aggressive, opportunistic fungal infection caused by one of the members of the mucoraceal family which include Absidia, Mucor, and Rhizopus (1,6,7). Mucor is a saprophytic fungus and it causes aggressive angioinvasive disease in immunocompromised and diabetic patients (3,7,8,9). However, Mucorales are found freely in the environment and are commonly seen in bread molds and decaying vegetation; they grow rapidly with constant discharge of spores into the environment (9). 

After inhalation or inoculation, fungi cause necrotizing vasculitis and rapidly extend into surrounding structures (6). This results from peri vascular, peri neural and soft tissue invasion by the fungus and causes suppurative arteritis, vascular thrombosis, and infarction of the surrounding tissue (6). The patient very often presents with acute headache, fever, and sometime neurological deficits as well (6). In our patient, the fungal mass was present medial to the lateral plate of the sigmoid sinus plate pressurizing the lumen of the sigmoid sinus causing clinical features of lateral sinus thrombosis.The ear and temporal bone are involved in invasive mucormycosis-type fungus spreads into the temporal bone by the tympanogenic, meningogenic, hematogenic, and nasopharyngeal route (9). Tympanogenic spread can occur when the primary focus is in the middle ear cleft of the temporal bone. Meningogenic temporal bone involvement follows meningitis via the internal auditory meatus, and the nasopharyngeal spread to the temporal bone via the Eustachian tube and fungaemia secondary to hematogenous spread of mycosis to temporal bone (9).

There are no clear evaluation methods to diagnose Mucormycosis and OM except tissue sampling, CT and MR imaging to obtain a provisional diagnosis (10). Histopatho- logically, the Mucor shows an acute or chronic inflammatory process with necrotic areas and fungal hyphal elements. The hyphae of Mucor are characteristically broad, ribbon-like with irregular branching at right angles (6,7,10) Histological differences between the Mucor and common Aspergillus fungus is empty appearing, nonseptate hyphae which are readily visible after routine hematoxylin and eosin staining (3,6,7).

Management of Mucormycosis is not well established but consists of three parts: Management of the underlying systemic and immunologic disease, surgical debridement of dead tissue, and anti-fungal therapy. Prolonged dosing of systemic amphotericin- B, voriconazole, posaconazole can achieve a good success rate. Control of predisposing conditions and hyperbaric oxygen can be used to control the disease (3,6,7,9).

## Conclusion

Fungal mucormycosis associated with OM of the temporal bone is very rare in normal individuals. Can occur in any individual without any risk factor for no immunity. It is an aggressive and potentially fatal infection which needs early diagnosis and management. Surgical debridement maybe needed in most of the cases.

## References

[1] Nomiya R, Nomiya S, Paparella MM (2008). Mucormycosis of the Temporal Bone. Otol Neurotol.

[2] Biniyam K, Bhat V, Kumar S, Bhandary B, Aroor R (2014). Asymptomatic mucormycosis of middle ear: An incidental finding during Tympanoplasty. Ind J Otol.

[3] Nirmala SVSG, Lalitha V, Sivakumar N, Kiran Kumar K, Srikanth M (2011). Mucormycosis associated with juvenile diabetes. J Ind Soc Pedodontics Prevent Dentistr.

[4] Alva B, Chandra Prasad K, Chandra Prasad S, Pallavi S (2009). Temporal bone osteomyelitis and temporoparietal abscess secondary to malignant otitis externa. J Laryngol Otol.

[5] Blyth CC, Gomes L, Sorrel TC, da Cruz M, Sud A, Chen SCA (2011). Skull-base osteomyelitis: fungal vs bacterial infection. Clin Microbiol Infect.

[6] Stodulski D, Kowalska B, Stankiewicz C (2006). Otogenic skull base osteomyelitis caused by invasive fungal infection Case report and literature review. Eur Arch Otorhinolaryngol.

[7] Aggarwal SK, Agarwal P (2015). Zygomycosis of temporal bone in uncontrolled diabetes mellitus: A rare cause for skull base osteomyelitis. Muller J Med Sci Res.

[8] Chan LL, Singh S, Jones D, Diaz Jr EM, Ginsberg LE (2000). Imaging of Mucormycosis Skull Base Osteomyelitis. AJNR Am J Neuroradiol.

[9] Kuruvilla G, Job A, Mathew J, Ayyappan AP, Jacob M (2006). Septate fungal invasion in masked mastoiditis: a diagnostic dilemma. J Laryngol Otol.

[10] Chang PC, Fischbein NJ, Holliday RA (2003). Central Skull Base Osteomyelitis in Patients without Otitis Externa: Imaging Findings. AJNR Am J Neuroradiol.

